# Supplementary material for: Uridine Diphosphate Glucuronosyl Transferase 2B28 (UGT2B28) Promotes Tumor Progression and Is Elevated in African American Prostate Cancer Patients
Source: Cells. 2022 Jul 29;11(15):2329. doi: 10.3390/cells11152329 (PMC9367340; doi:10.3390/cells11152329)
Supplement: Supplementary file 1 [file cells-11-02329-s001.zip › Cells_Supplementary Files_FINAL_CORRECTED.pdf]

Supplementary Table S1: List of primers used.

Primer	Sequence
UGT2B28	F: 5'- GTGATTGGGTTTCTGCTGGC - 3'<br>R: 5'- TGATGTCAACCCATCTATCTGGT - 3'
UGT2B11	F: 5'- CCAAACGTTGATTTTGTTGGA - 3'<br>R: 5'- TCTGTACAAACTCCTCCATTTCC - 3'
UGT2B15	F: 5'- ACTTTAGGTTCCAATACTCGACTG - 3'<br>R: 5'- CGCCTCATAGATGCCATTGGTTC - 3'
UGT2B17	F: 5'- ACCAGCCAAACCCTTGCCTAAG - 3'<br>R: 5'- GGCTGATGCAATCATGTTGGCAC - 3'
UGT2B10	F: 5'- GAAATGGACTACAGTTCTGCTGA - 3'<br>R: 5'- GTGGATGAGTCGTTGGGATCA - 3'
AR	F: 5'- CAAATCACCCCCCAGGAAT - 3'<br>R: 5'- CACTGGAATAATGCTGAAGAGTAGCA - 3'
PSA	F: 5'- CCTGTCCGTGACGTGGAT - 3'<br>R: 5'- CAGGGTTGGGAATGCTTC - 3'
18s	F: 5'- GCAGAATCCACGCCAGTACAAG - 3'<br>R: 5'- GCTTGTTGTCCAGACCATTGGC - 3'
β-actin	F: 5'- CACCATTGGCAATGAGCGGTTC - 3'<br>R: 5'- AGGTCTTTGCGGATGTCCACGT - 3'

Supplementary Table S2: List of MRM transitions used for measurement of UDP-glucuronic acid

Compound name	Precursor ion	Product ion	Polarity
Glucose	179.0	59.0	Negative
[13C]6 Glucose	185.1	61.0	Negative
UDP-glucuronic acid	579.0	78.9	Negative
UDP glucuronic acid[13C]6	585.0	78.9	Negative

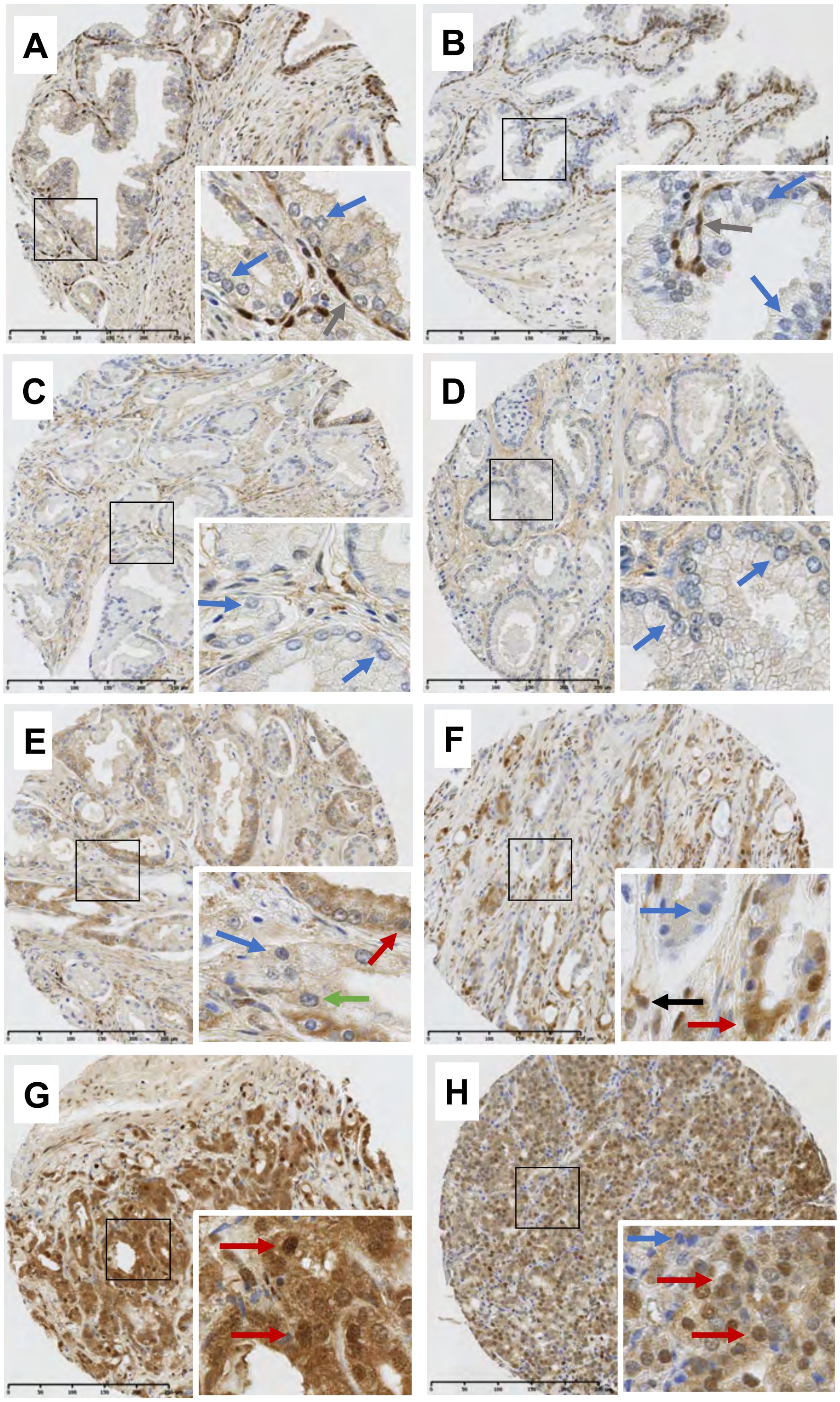

Supplemental Figure S1. Example of variable expression of UGT2B28 observed in the prostate of African American (A,C,E,G) and European American (B,D,F,H) patients. A) and B) UGT2B28 in benign glands presented nuclear staining in basal cells (blue arrows) with variable staining of nuclear secretory cells. In these two examples, prostatic secretory cells are negative for UGT2B28 (blue arrows). C) and D) Tumors with negative expression of UGT2B28 indicated by blue arrows, and in E) to H) tumors with variable nuclear and cytoplasmic UGT2B28 staining expression. The blue arrows show the negative and the black arrows the positive UGT2B28 nuclear staining and red arrows illustrate expression in both nuclear and cytoplasmic compartments and tumor samples with only cytoplasmic staining are indicated by green arrows. E) and F) 50% and G) and H) >90% of nuclear and cytoplasmic UGT2B28 staining expression. An enlargement is available to better visualize the different nuclear or cytoplasmic expression of UGT2B28.

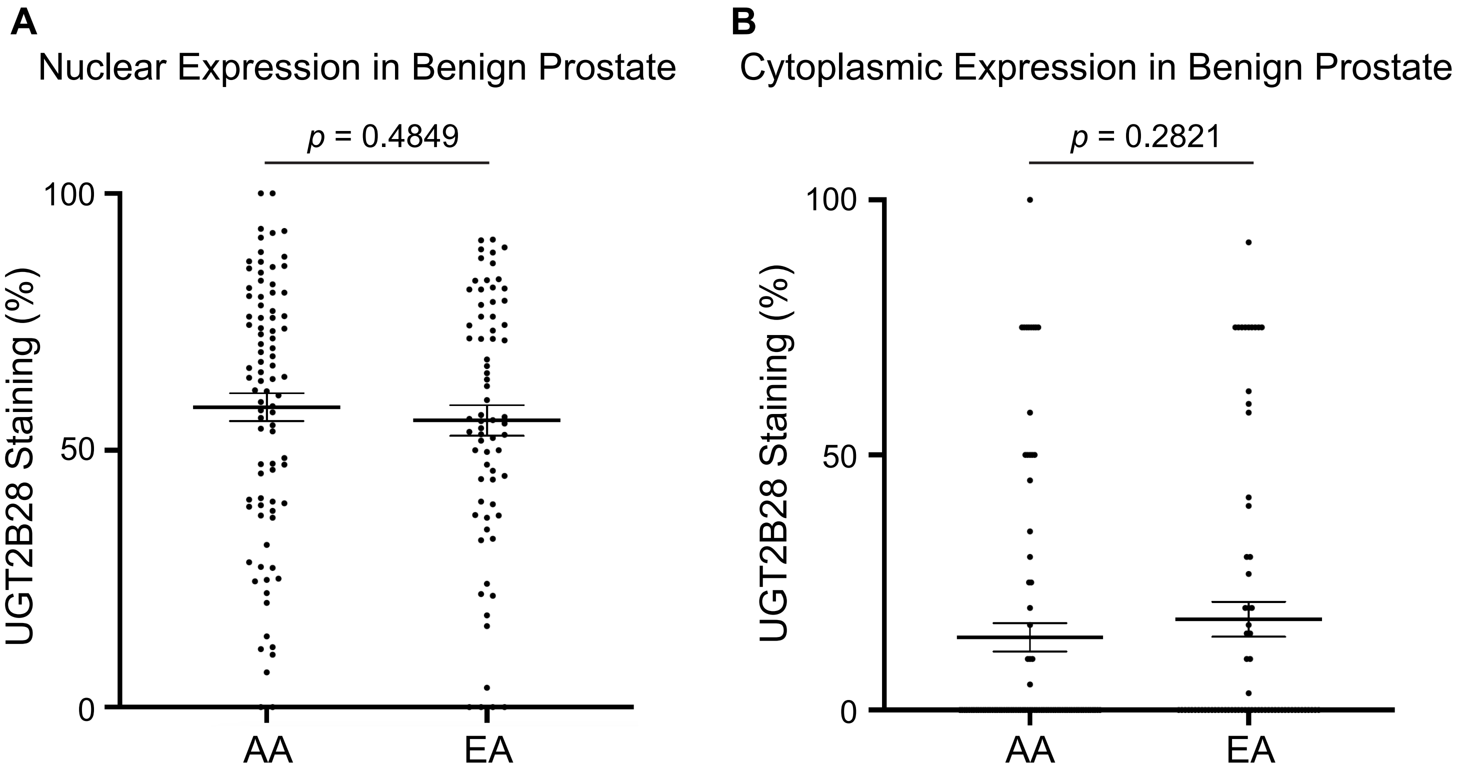

Supplementary Figure S2. Unaltered UGT2B28 protein expression in African American (AA) vs. European American (EA) benign adjacent prostate tissues. **A)** Percent nuclear staining for UGT2B28 is unaltered in African American vs. European American benign adjacent prostate tissues. **B)** Percent cytoplasmic staining for UGT2B28 is unaltered in African American vs. European American benign adjacent prostate tissues.

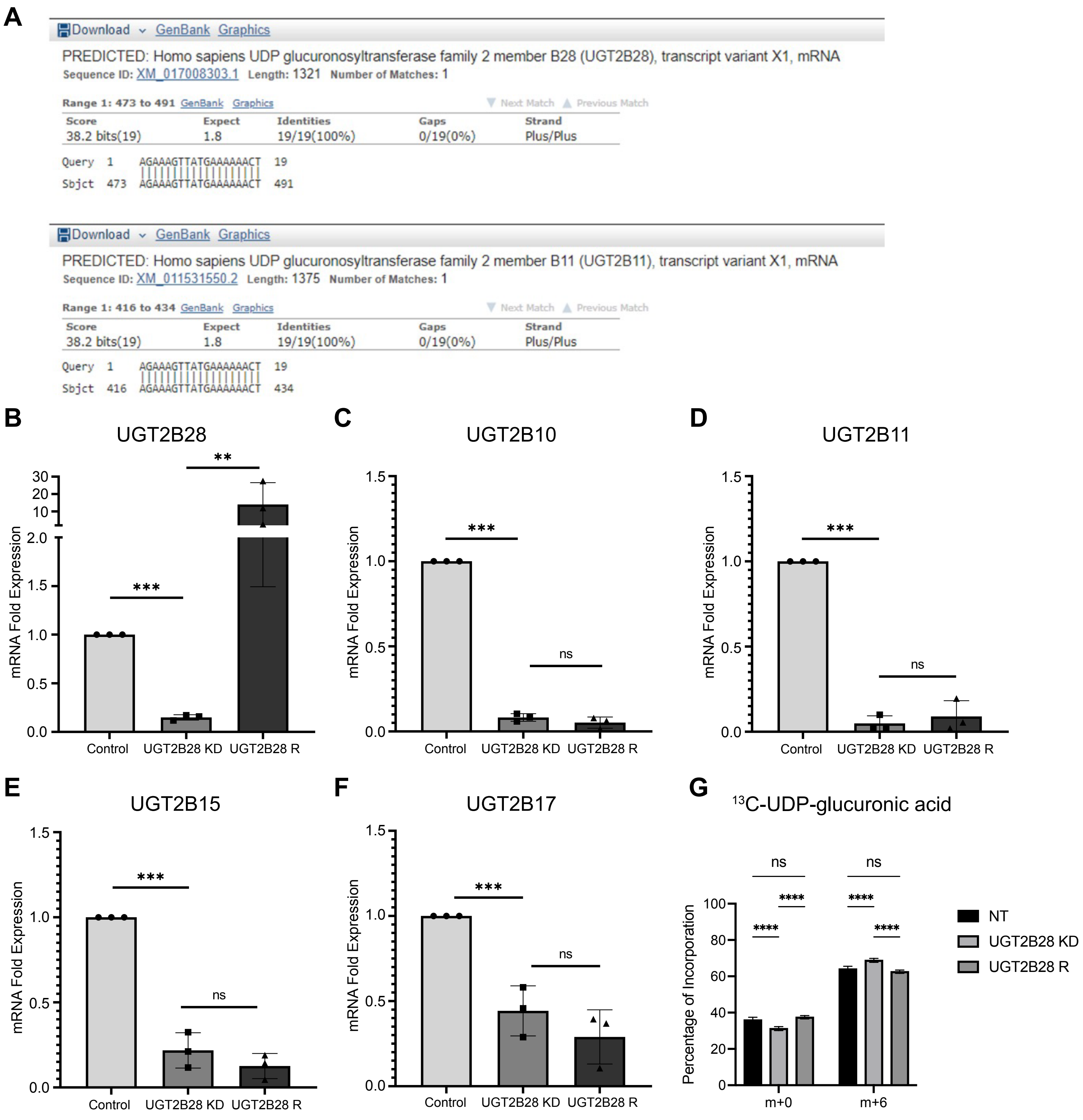

Supplemental Figure S3. Specificity of UGT2B28 rescue in androgen dependent cell lines. **(A)** NCBI BLAST results of the shRNA sequence for KD4. Transcript levels of **(B)** UGT2B28 **(C)** UGT2B10 **(D)** UGT2B11 **(E)** UGT2B15 and **(F)** UGT2B17 in response to UGT2B28 KD with KD4 sh-RNA clone and re-expression rescue using UGT2B28 cDNA. Only UGT2B28 expression was rescued in the re-expression setting. **(G)** [13C]-UDP-glucuronic acid levels in NT-, UGT2B28 KD-, and UGT2B28 R-transduced LNCaP cells. **$p<0.001$, ***$p<0.0001$, n=3. Cells were labeled with universal-ly labeled uniformly labeled [^{13}C]-glucose for 24h and the levels of [^{13}C]-UDP-glucuronic acid were measured in the cell pellets as described in the methods section. ****$p<0.00001$, n=4, Two-way Anova.

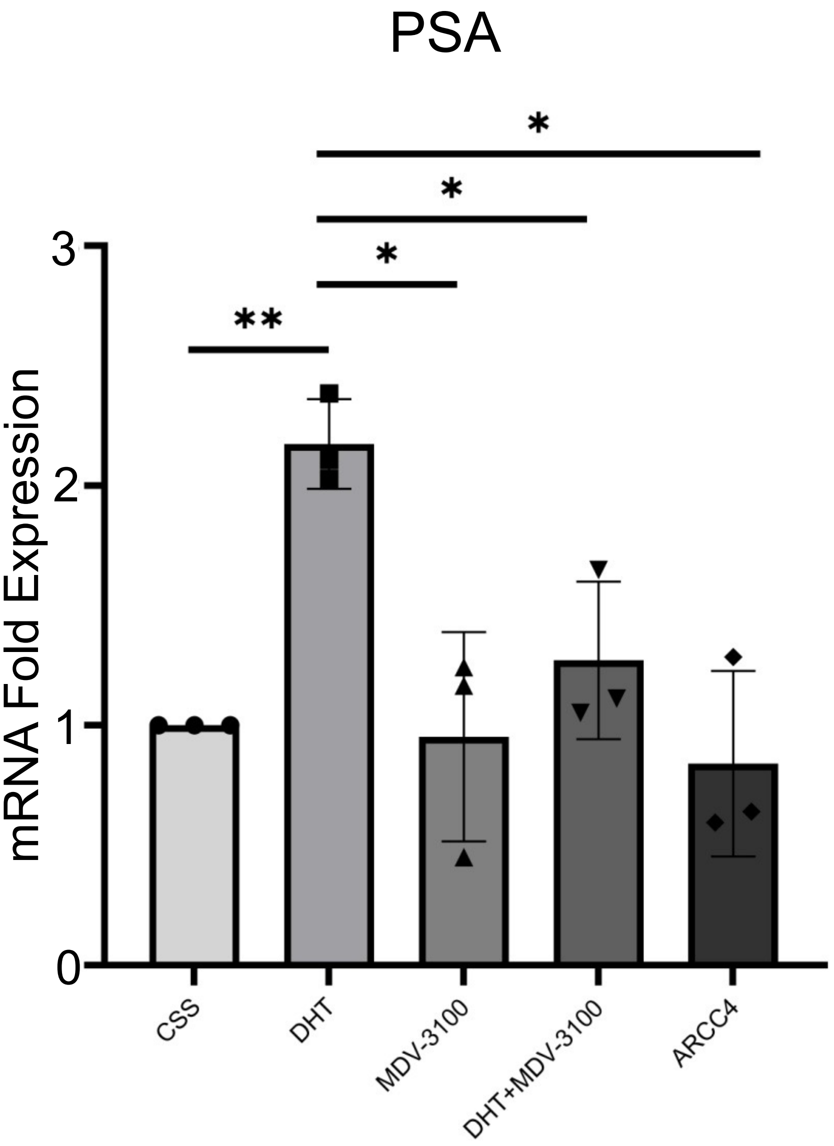

Supplemental Figure S4. PSA transcript levels in LNCaP cells treated with 1nM 5α-dihydrotestosterone (DHT), 10μM MDV-3100, 1nM DHT+10μM MDV-3100, and 300nM ARCC4 for 24h. *$p<0.01$, **$p<0.001$, $n=3$.

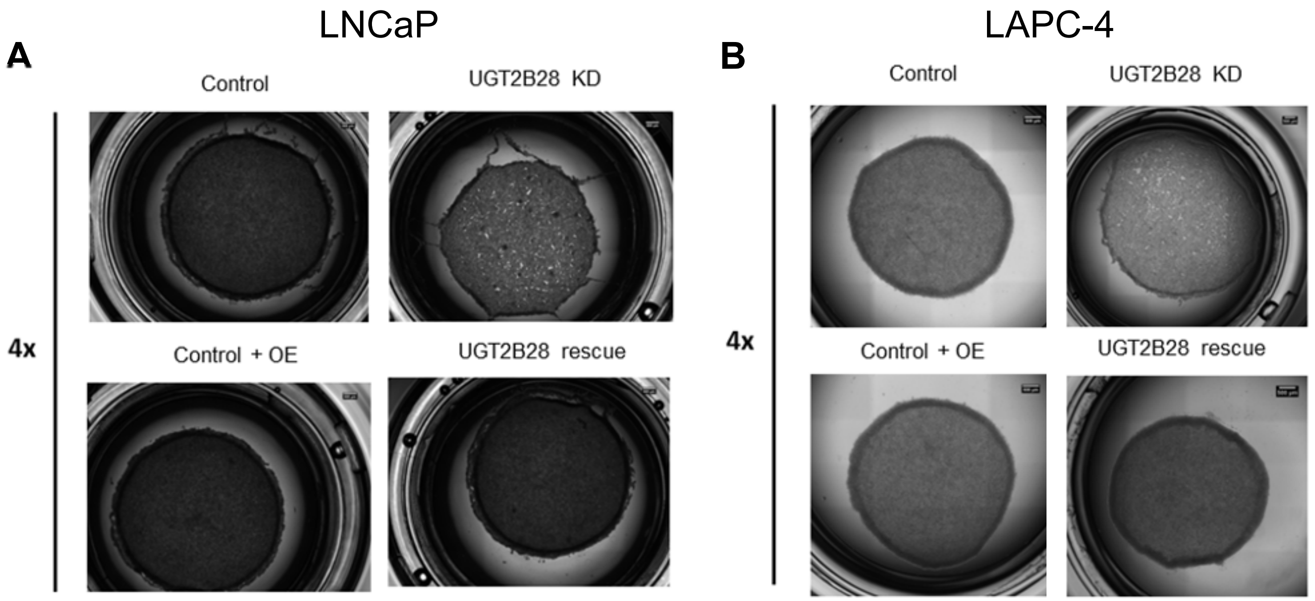

Supplemental Figure S5. 4x Bright-field images of (**A**) LNCaP and (**B**) LAPC4 organoids transduced with control (NT), UGT2B28 KD, UGT2B28 overexpression (Control + OE), and UGT2B28 re-expression in the KD setting (UGT2B28 rescue) grown on Millicell inserts at 24 h. n = 3. Scale bar = 500 μm.

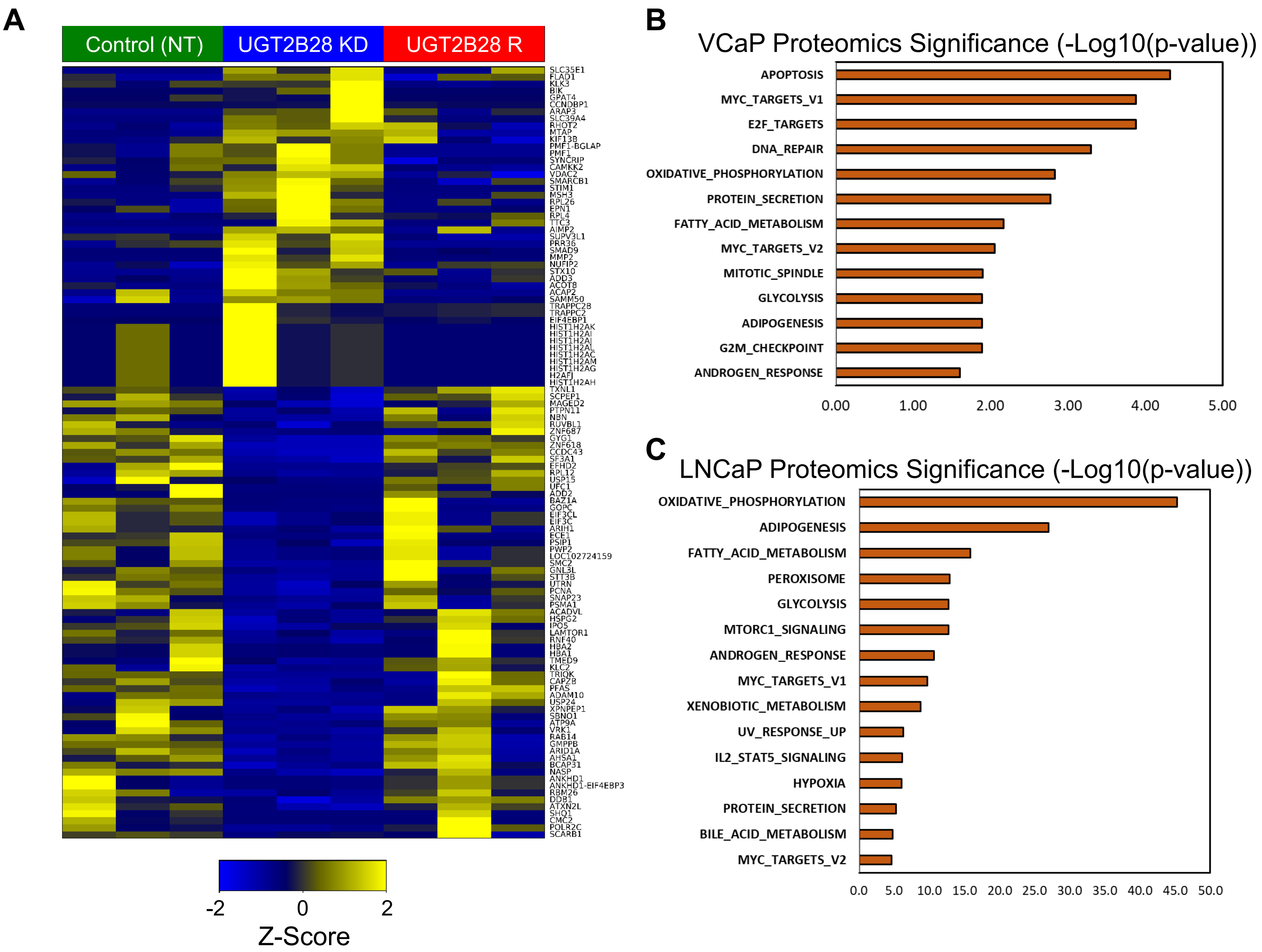

Supplemental Figure S6. Common pathways are altered in LNCaP cells and VCaP tumors. A. Heat map showing mass spectrometry-based proteomics alterations in VCaP tumors containing control (NT), UGT2B28 KD and UGT2B28 R in intact mice. Refer to the scale bar for expression levels. B. Pathway analysis of proteins in VCaP tumors significantly altered upon UGT2B28 KD vs. control (NT) and rescued with UGT2B28 overexpression (UGT2B28 R). C. Pathway analysis of proteins in LNCaP cells significantly altered upon UGT2B28 KD vs. control (NT) and rescued with UGT2B28 overexpression (UGT2B28 R).
